# Primary splenic angiosarcoma initially diagnosed as littoral cell angioma: A case report of a diagnostic pitfall

**DOI:** 10.1097/MD.0000000000051451

**Published:** 2026-11-06

**Authors:** Yi Yu, Hong Zhang, Gaosong Zhang, Liying Xu

**Affiliations:** a The First Clinical Medical College, Zhejiang Chinese Medical University, Hangzhou, China; b Department of Respiratory Medicine, The First Affiliated Hospital of Zhejiang Chinese Medical University (Zhejiang Provincial Hospital of Chinese Medicine), Hangzhou, China; c Department of Gastroenterology, People’s Hospital of Xinchang, Shaoxing, China; d Department of Medical Administration, The First Affiliated Hospital of Zhejiang Chinese Medical University (Zhejiang Provincial Hospital of Chinese Medicine), Hangzhou, China.

**Keywords:** case report, diagnostic pitfall, littoral cell angioma, primary splenic angiosarcoma, suspected hepatic metastases

## Abstract

**Rationale:**

Primary splenic angiosarcoma (PSAn) is a rare and aggressive vascular malignancy that poses diagnostic challenges because of its overlapping morphological and immunohistochemical features with benign splenic vascular tumors.

**Patient concerns:**

A 71-year-old woman presented with left upper abdominal pain, severe anemia, thrombocytopenia, and a splenic lesion. Splenectomy and excision of a separate 1-cm hepatic nodule were performed, with initial pathological diagnoses of splenic littoral cell angioma and hepatic cavernous hemangioma. Persistent postoperative fever, progressive cytopenias, jaundice, hepatic dysfunction, and newly developed diffuse multifocal hepatic lesions subsequently occurred.

**Diagnoses:**

Pathological reevaluation of the splenic specimen using an expanded immunohistochemical panel resulted in a revised diagnosis of PSAn. The subsequently developed diffuse hepatic lesions were considered highly suspicious for hepatic metastases based on the radiological and clinical findings, although histological confirmation was unavailable.

**Interventions:**

The patient underwent splenectomy and excision of the initial hepatic nodule. Multidisciplinary reassessment and pathological reevaluation were performed following clinical deterioration. The patient declined liver biopsy and further oncological treatment.

**Outcomes:**

Despite supportive management, the patient’s condition progressively deteriorated. She was discharged after 2 weeks and died 2 months later.

**Lessons:**

PSAn should be considered in the differential diagnosis of splenic vascular tumors, particularly when morphological and immunohistochemical findings overlap. Unexpected postoperative deterioration should prompt timely multidisciplinary reassessment and pathological review.

## 1. Introduction

Primary splenic angiosarcoma (PSAn) originates from immature endothelial-type cells of the splenic blood vessels. It is a rare and aggressive malignant neoplasm with a high rate of metastasis (70–85%) and a poor prognosis (median overall survival of only 8 months). It has a reported frequency of only 0.14 to 0.25 cases per million persons annually and is among the rarest of all neoplasms.^[1]^ We report on a 71-year-old woman whose initial splenic diagnosis of littoral cell angioma (LCA) was revised to PSAn after persistent postoperative fever, progressive cytopenias, hepatic dysfunction, and the development of diffuse hepatic lesions that were radiologically suspicious for metastases. A brief account of this case was previously reported.^[2]^ In the present analysis, we revisit the case with a focused comparison between PSAn and LCA, examine the morphologic and immunophenotypic factors that contributed to the initial diagnosis, and integrate the serial clinical, laboratory, and imaging findings to identify practical triggers for pathology rereview when the postoperative course is inconsistent with an initially benign diagnosis. This case report was prepared in accordance with the Case Report guidelines.^[3]^

## 2. Presentation of case

The chronological sequence of the patient’s clinical course, investigations, pathological reassessment, and outcomes is summarized in Supplemental Digital Content 1. A 71-year-old woman was admitted to a county-level hospital with a 12-day history of left upper quadrant abdominal pain after a fall. She reported left-sided chest pain before the fall and localized swelling afterward; the pain gradually worsened and radiated to the left upper abdomen. Her medical history included hypertension for 2 years. She reported no regular medication use, known drug allergies, relevant family history, smoking, or alcohol consumption. On examination, her body temperature was normal and blood pressure was 160/90 mm Hg. Physical examination revealed tenderness over the left chest wall and in the left upper quadrant of the abdomen.

Laboratory tests on admission revealed a white blood cell count of 10.71 × 10^9^/L, neutrophil percentage of 75.4%, hemoglobin 60 g/L, and platelet count 69 × 10^9^/L. Abdominal ultrasonography demonstrated marked splenomegaly with suspected intraparenchymal hemorrhage. The radiological findings obtained during the initial evaluation and subsequent readmission are presented in Figure 1A to C. Unenhanced computed tomography (CT) revealed a round, hyperattenuating splenic lesion measuring 25 × 22 mm, with an attenuation value of approximately 70 HU. During the portal venous phase, the lesion appeared relatively hypoattenuating compared with the surrounding splenic parenchyma (Fig. 1A, panels a–c). These findings suggested a hemorrhagic vascular splenic lesion but did not permit reliable differentiation among PSAn, LCA, and other splenic vascular tumors. No diffuse multifocal hepatic lesions were identified on the preoperative CT.

**Figure 1.**
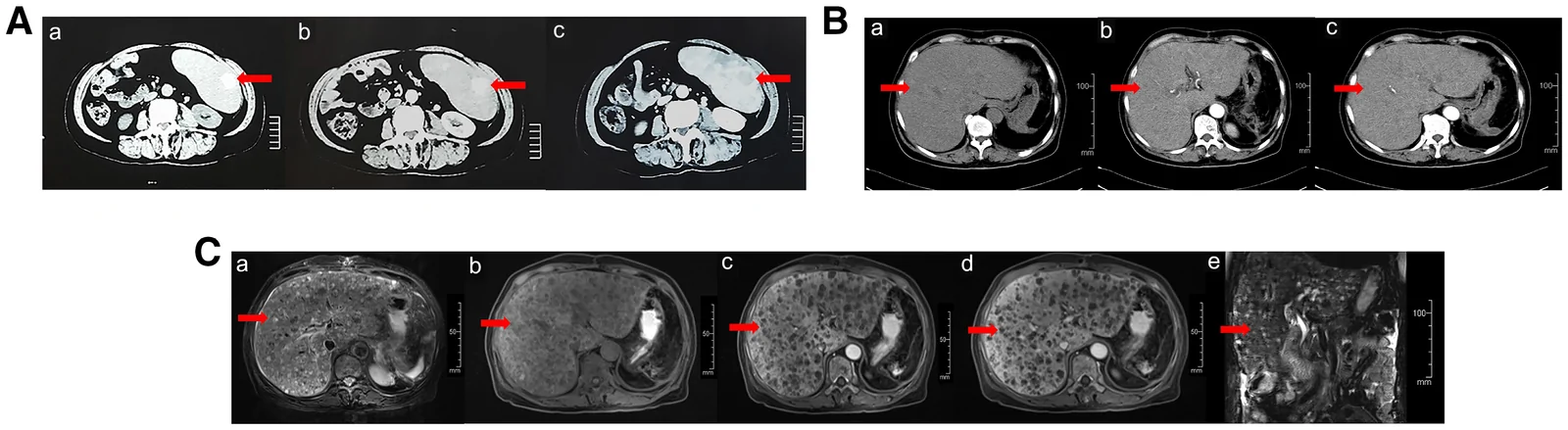
Radiological findings. (A) Initial abdominal computed tomography findings. CT performed at the referring hospital demonstrated marked splenomegaly and a round, hyperattenuating splenic lesion measuring 25 × 22 mm, with an attenuation value of approximately 70 HU on the unenhanced image. During the portal venous phase, the lesion appeared relatively hypoattenuating compared with the surrounding splenic parenchyma. (a) Plain CT image. (b) Enhanced CT image obtained during the hepatic artery phase. (c) Enhanced CT image obtained during the portal vein phase. The red arrows show the splenic space-occupying lesion. (B) Follow-up abdominal computed tomography findings at readmission. Contrast-enhanced CT demonstrated marked hepatomegaly with numerous small nodular lesions diffusely involving the liver. The rapid development and diffuse distribution of these lesions raised strong radiological suspicion of hepatic metastases. Multiple small hypoattenuating lesions in both kidneys were consistent with simple renal cysts. Small-volume pelvic ascites and postoperative changes in the splenic bed were also observed. (a) Plain CT image. (b) Enhanced CT image obtained during the hepatic artery phase. (c) Enhanced CT image obtained during the portal vein phase. The red arrows show the multiple space-occupying lesions in the liver. (C) Follow-up abdominal magnetic resonance imaging and magnetic resonance cholangiopancreatography findings. MRI demonstrated marked hepatomegaly with diffuse multifocal hepatic nodules. The lesions were hypointense on T1-weighted images and showed heterogeneous signal intensity on T2-weighted images, ranging from mildly hypointense to hyperintense, without appreciable postcontrast enhancement. MRCP showed normal-caliber intrahepatic and extrahepatic bile ducts without evidence of biliary obstruction. Postsplenectomy changes, mild pleural effusion, and small-volume ascites were also noted. (a) Plain MRI scan. (b) Enhanced MRI scan obtained during the hepatic artery phase. (c) Enhanced MRI scan obtained during the portal vein phase. (d) Enhanced MRI scan obtained during the hepatic venous phase. (e) MRCP. The red arrows show the multiple space-occupying lesions of the liver. CT = computed tomography, MRCP = magnetic resonance cholangiopancreatography, MRI = magnetic resonance imaging.

An open splenectomy and resection of a left hepatic tumor were performed 3 days later. The spleen was 16 cm × 12 cm × 5 cm with an intact capsule. The liver appeared normal except for a 1-cm tumor at the left lobe margin. The pathological findings were LCA of the spleen and cavernous hemangioma of the liver (Fig. 2A, panels a–d). Immunohistochemical staining of the splenic specimen was positive for CD31 and CD34, negative for CD20, CD56, epithelial membrane antigen, CD3 and cytokeratins, while the Ki-67 proliferation index was <10%.

**Figure 2.**
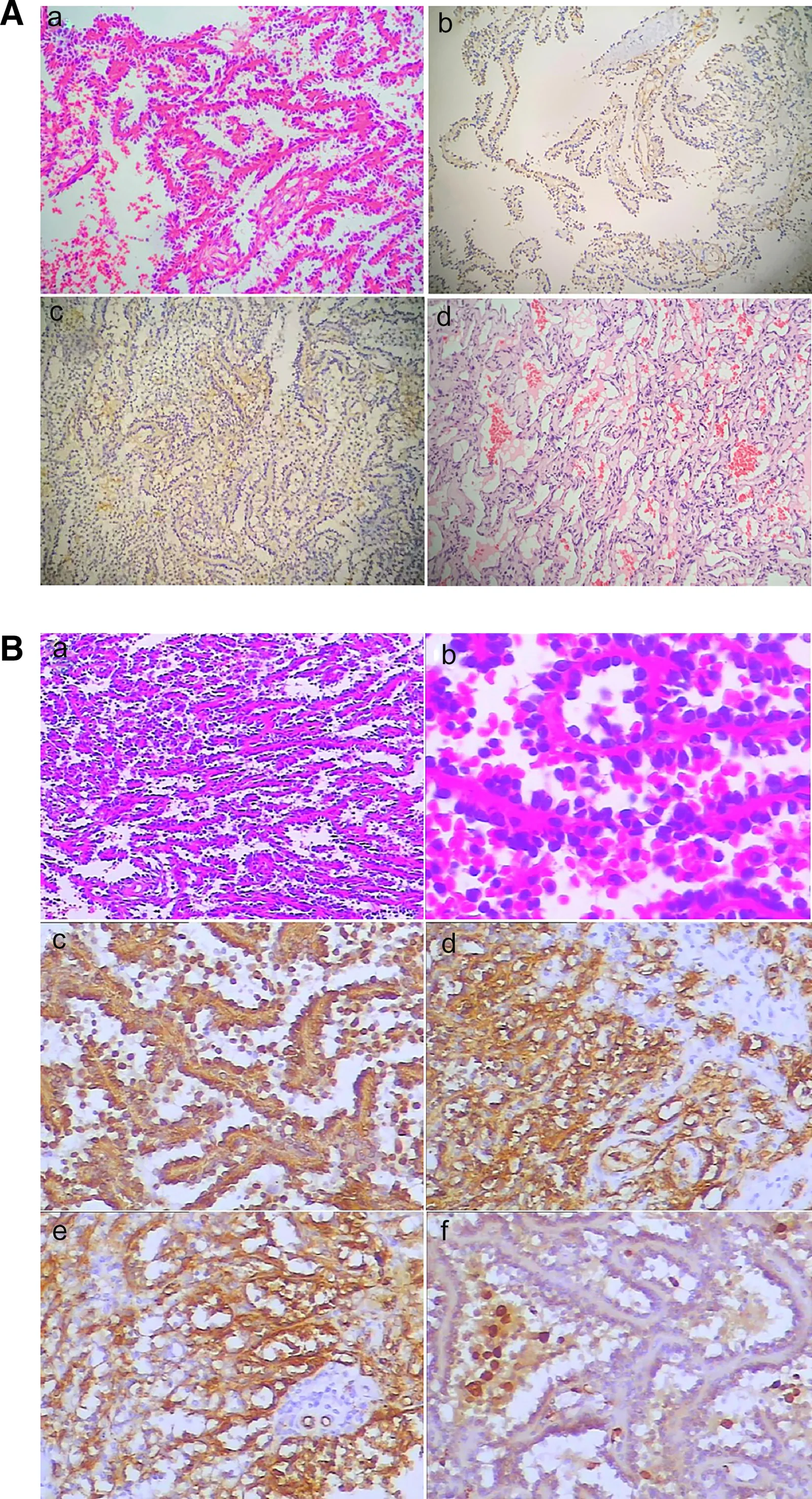
Histopathological findings. (A) Histopathological examination performed at the previous hospital initially led to diagnoses of splenic LCA and hepatic cavernous hemangioma. (a) HE staining (×200) of the spleen showing reduced cytoplasm in certain regions. The nuclei are ovoid with light staining, morphologically resembling normal splenic sinus cells. (b, c) Immunohistochemical staining demonstrating positive expression of CD31 and CD34 (both × 200). (d) HE staining (×200) of the liver. (B) Histopathological findings of the splenic specimen. (a) HE staining (×200). (b) HE staining (×400). HE staining demonstrates the proliferation and interconnection of neoplastic endothelial cells forming tube-like structures, some of which are papillary. Red blood cells and foam cells are observed within the interstitial areas, along with hemorrhage, necrosis, and fibrous tissue hyperplasia. The tumor cells are cuboidal or columnar, exhibiting large hyperchromatic nuclei, marked atypia, and occasional mitotic figures. (c–f) Immunohistochemical staining demonstrating positive expression of F8, CD34, CD31, and CD68, respectively (all × 400). HE = hematoxylin and eosin; LCA = littoral cell angioma.

After surgery, the patient developed a persistent fever for more than 2 months, accompanied by right upper quadrant abdominal pain, fatigue, and poor appetite. Although the pain was tolerable and broad-spectrum intravenous antibiotics provided transient symptomatic improvement, her overall condition continued to deteriorate. She was admitted to the emergency department of a tertiary referral hospital 3 months after surgery.

The physical examination on presentation revealed a mild fever (37.6°C), high blood pressure (169/80 mm Hg), jaundice, hepatomegaly, anemia-related signs, and postoperative abdominal scars. Laboratory examinations revealed a white blood cell count of 3.6 × 10^9^/L, red blood cell count of 2.89 × 10^12^/L, hemoglobin 79 g/L, platelet count 20 × 10^9^/L, and C-reactive protein 26 mg/L. Additional tests demonstrated jaundice, coagulopathy, and significantly impaired hepatic function (Table 1A). Serum tumor markers, such as α-fetoprotein, carcinoembryonic antigen, carbohydrate antigen 19-9, and carbohydrate antigen 125, were all within normal limits.

**Table 1:** Laboratory findings and main clinicopathological differences between primary splenic angiosarcoma and littoral cell angioma.

A. Results of blood examinations				
A1. Bilirubin test (μmol/L)				
Date	TBIL	DBIL	IBIL	
June 12	81.7	45.5	36.2	
June 15	96.7	67.6	29.1	
June 20	213.4	142.1	71.3	
June 25	289	213.8	75.2	
A2. Liver function test (U/L)				
Date	ALP	GGT	ALT	AST
June 12	657	324	119	156
June 15	593	320	109	163
June 20	592	324	97	188
June 25	466	223	37	76
A3. Coagulation function test				
Date	D-D (mg/L)	PT (s)	FIB (g/L)	
June 12	45.53	12.5	1.96	
June 15	50.87	13.3	1.82	
June 20	54.99	14.8	1.69	
June 25	56	15.7	0.96	
B. Main clinicopathological differences between PSAn and LCA				
Feature	LCA	Malignant littoral-cell neoplasms (LCHE/LCAS)		PSAn
Biological behavior	Usually benign; recurrence or metastasis is not expected.	Malignant potential; recurrence or metastasis may occur after a prolonged interval, although aggressive courses are also reported.		Highly aggressive, with frequent early hematogenous dissemination.
Background architecture	Anastomosing sinus-like channels and cyst-like spaces with papillary fronds; lining cells may detach into the lumina.	Extensive areas resembling conventional LCA, accompanied by solid, spindle-cell, clear-cell, infiltrative, or necrotic components.		Heterogeneous malignant vascular proliferation with irregular anastomosing channels and papillary, solid spindle-cell, or epithelioid areas.
Cytologic and growth features	Bland lining cells, minimal atypia, and low mitotic activity; destructive invasion is absent.	Atypia and mitotic activity are variable and may be subtle; solid or infiltrative growth, necrosis, or metastasis indicates malignant potential.		Enlarged hyperchromatic endothelial cells, marked atypia, mitotic activity, and destructive or infiltrative growth; well-differentiated areas may appear deceptively bland.
Hemorrhage and necrosis	Extensive destructive hemorrhage and necrosis are not typical.	May occur, particularly within malignant components.		Common and often associated with marked intratumoral heterogeneity.
Erythrophagocytosis	Frequently observed and supportive of littoral-cell differentiation.	May be retained in LCA-like areas but is not invariably present.		Not a defining feature; histiocytic differentiation may nevertheless occur.
Typical immunophenotype	Dual endothelial–histiocytic phenotype: CD31+, CD68/CD163+, and often CD21+; typically CD34- and CD8-.	Usually retains a dual endothelial–histiocytic phenotype similar to LCA: CD31 and CD68/CD163 are commonly expressed, CD21 may be retained, and CD34/CD8 are usually negative in reported cases.		Endothelial markers such as CD31, ERG, FLI1, factor VIII, and variably CD34 are expressed. CD68 or lysozyme may also be positive, but this does not establish littoral-cell differentiation.

(A1) Bilirubin results indicate jaundice. (A2) Liver function indices indicate marked hepatic dysfunction; their trend was opposite to that of bilirubin levels. (A3) Coagulation results indicate worsening coagulopathy. (B) No single immunohistochemical marker reliably distinguishes these entities. The patterns listed above are typical rather than absolute.

ALP = alkaline phosphatase, ALT = alanine aminotransferase, AST = aspartate aminotransferase, DBIL = direct bilirubin, D-D = D-dimer, FIB = fibrinogen, GGT = gamma-glutamyl transferase, IBIL = indirect bilirubin, LCA = littoral cell angioma, LCAS = littoral cell angiosarcoma, LCHE = littoral cell hemangioendothelioma, PSAn = primary splenic angiosarcoma, PT = prothrombin time, TBIL = total bilirubin.

At readmission, contrast-enhanced CT demonstrated marked hepatomegaly with numerous nodular lesions diffusely distributed throughout the liver (Fig. 1B, panel a). The hepatic lesions showed only mild enhancement after contrast administration, and the spleen was absent following splenectomy (Fig. 1B, panels b–c). Magnetic resonance imaging and magnetic resonance cholangiopancreatography confirmed diffuse multifocal hepatic nodules without appreciable enhancement. No dilatation or obstruction of the intrahepatic or extrahepatic bile ducts was observed (Fig. 1C, panels a–e). The rapid interval development of these lesions, together with the revised diagnosis of PSAn and progressive hepatic dysfunction, was considered highly suspicious for hepatic metastases; however, histological confirmation was unavailable.

Hemoglobin decreased to 70 g/L and platelet count decreased to 14 × 10^9^/L. Albumin decreased to 27.8 g/L. Routine urinalysis revealed occult blood 2+, bilirubin 2+, and red blood cell 2+. The available infectious, autoimmune, and hematologic investigations did not identify an alternative explanation for the persistent fever. Bilirubin levels increased continuously, while hepatic and coagulation functions deteriorated progressively during hospitalization (Table 1A). Bone marrow aspiration was performed 5 days after hospitalization and the results showed poor megakaryocyte function.

However, the patient still complained of abdominal distention, pain, and a persistent low fever (37.5–38.2°C). A multidisciplinary team discussion of this intractable case led to a suspected diagnosis of splenic angiosarcoma with hepatic metastasis. At that time, the available pathological sections were rereviewed by the pathology department of the tertiary hospital. The rereview demonstrated malignant vasoformative morphology and was reported as PSAn. Immunohistochemical staining was positive for Vimentin, CD31, CD34, and factor VIII; focal positivity for CD68 and CD8; negative for smooth muscle actin, P53 and cytokeratin; while the Ki-67 proliferation index was 20 % (Fig. 2B, panels a–f).

After multidisciplinary discussion, the diffuse hepatic lesions were considered radiologically highly suspicious for metastases. The patient declined liver biopsy; therefore, metastatic involvement remained unconfirmed histologically. After counseling regarding the diagnostic uncertainty, prognosis, and available management options, she declined further oncologic treatment and was discharged after 2 weeks. Unfortunately, she died 2 months later.

## 3. Clinical discussion

Fewer than 300 cases of PSAn have been reported since it was first described in 1879 by Langhans.^[1,4]^ Because clinical manifestations are nonspecific and imaging findings can overlap with benign splenic vascular lesions, PSAn is frequently misdiagnosed, leading to delayed recognition and poor outcomes.^[1,5]^ In the present case, the initial pathological diagnosis following splenectomy was LCA. However, after the patient experienced rapid clinical deterioration, pathological review led to a revised diagnosis of PSAn.

Previous reports relevant to this diagnostic pitfall can be broadly divided into 2 distinct but complementary groups (Supplemental Digital Content 2). The first comprises cases of PSAn initially misinterpreted as benign splenic vascular tumors. Xu et al^[6]^ reported a case initially diagnosed as splenic hemangioma. Two years after splenectomy, the patient developed hepatic and osseous metastases, and comparison of the metastatic lesions with the original splenic specimen ultimately led to a revised diagnosis of well-differentiated PSAn. Cao et al^[7]^ similarly described a patient whose splenic lesion was initially diagnosed as a hemangioma but was followed by metastatic disease after splenectomy. These cases illustrate how well-differentiated or morphologically heterogeneous PSAn may be mistaken for a benign vascular lesion.

The second group consists of malignant tumors of littoral cell origin, including littoral cell hemangioendothelioma and littoral cell angiosarcoma. Although these entities differ from conventional PSAn, they are directly relevant to the differential diagnosis in the present case. The malignant littoral cell tumors reported by Ben-Izhak et al,^[8]^ Fernandez et al,^[9]^ Larsen et al,^[10]^ and Wang et al^[11]^ all contained extensive LCA-like areas; however, solid, spindle-cell, infiltrative, necrotic, or otherwise atypical components indicated malignant potential. These reports demonstrate that an LCA-like architecture or a dual endothelial–histiocytic immunophenotype alone does not establish benign biological behavior. The principal features distinguishing these 3 entities are summarized in Table 1B.

Taken together, these reports reflect a broader diagnostic challenge across the spectrum of splenic vascular tumors rather than a single mechanism of diagnostic delay unique to PSAn. Splenomegaly, cytopenias, nonspecific imaging findings, intratumoral morphological heterogeneity, and overlapping expression of endothelial and histiocytic markers may occur in several of these entities. CD31 positivity alone cannot distinguish LCA from PSAn. Splenic angiosarcoma may also express CD68,^[1,12]^ and PSAn can show concurrent expression of endothelial and histiocytic markers. Accurate classification therefore requires adequate sampling and an integrated assessment of vascular architecture, cytological atypia, growth pattern, hemorrhage or necrosis, proliferative activity, and a sufficiently broad immunohistochemical panel.

The slides initially examined at the referring hospital showed relatively bland, sinusoid-like vascular areas within the spleen. Pathological review after the patient’s clinical deterioration revealed irregular, anastomosing, and focally papillary vascular channels lined by cuboidal or columnar endothelial cells. The tumor cells had enlarged, hyperchromatic nuclei with conspicuous cytological atypia and occasional mitotic figures, accompanied by hemorrhage and necrosis. Immunohistochemical staining was positive for the endothelial markers CD31, CD34, and factor VIII, and CD68 positivity was also reported. Taken together, these findings were considered most consistent with PSAn. Nevertheless, the discrepancy between the relatively bland LCA-like areas observed initially and the overtly malignant components identified on review may reflect intratumoral heterogeneity and sampling bias within a PSAn, but it may also raise the possibility of a malignant tumor of littoral cell origin. Because the archived slides and paraffin blocks were no longer available, additional multiregional sampling, confirmation of the cellular localization of CD68 staining, and further evaluation with littoral cell-associated markers could not be performed. The latter possibility, therefore, cannot be completely excluded.

Spontaneous splenic rupture is an important clinical manifestation of PSAn and is associated with an unfavorable prognosis. Previous studies suggest that patients who undergo splenectomy before rupture may survive longer than those treated after rupture. Splenectomy remains the principal treatment for resectable PSAn or disease confined to the spleen.^[5,13]^ In the present case, the splenic lesion was detected during evaluation after a fall. Initial imaging suggested possible intraparenchymal splenic hemorrhage but showed no definite capsular disruption or intraperitoneal bleeding; therefore, this case should not be classified as spontaneous splenic rupture. Nevertheless, splenomegaly accompanied by a hemorrhagic lesion remains an important warning sign of an aggressive splenic vascular neoplasm.

Imaging can support suspicion but is rarely diagnostic. Contrast-enhanced CT is useful for detecting splenic masses and rupture-related hemorrhage; however, findings can be heterogeneous and may mimic hemangioma or other benign vascular lesions. Magnetic resonance imaging may better characterize vascular tumors and necrosis patterns, while ^18^F-fluorodeoxyglucose positron emission tomography/CT can assist in staging metastatic spread and assessing treatment response.^[14,15]^ In our case, the initial CT appearance was atypical due to heterogeneous density, contributing to diagnostic uncertainty, and advanced imaging was not performed at first presentation. When PSAn is suspected, a multimodality approach may improve detection of metastatic disease and guide management decisions.

The patient’s rapid postoperative deterioration could not, by itself, distinguish PSAn from a malignant littoral cell tumor. Its importance lies in the marked inconsistency between the clinical course and the expected behavior of a purely benign LCA. When unexplained persistent fever occurs after splenectomy, routine evaluation for infection and postoperative complications should be undertaken first. However, when fever is accompanied by progressive cytopenias, worsening liver function, and the rapid appearance of multiple hepatic lesions, an underlying aggressive splenic vascular malignancy should be suspected, prompting timely clinicopathological review of the original diagnosis.

PSAn has an extremely poor prognosis because of its propensity for early hematogenous dissemination. Reported metastatic rates range from approximately 70% to 100%, with the liver and lungs being the most common sites.^[5]^ Although no liver biopsy was performed in this case, the rapid progression of diffuse hepatic lesions together with progressive deterioration of liver function was highly suggestive of metastatic disease. Evidence supporting systemic treatment for advanced PSAn remains limited.^[16,17]^ Because the patient declined further antitumor therapy, the effectiveness of such treatment could not be assessed in this case. Her death shortly after discharge was consistent with the fulminant clinical course that may follow metastatic dissemination of PSAn.

## 4. Strengths and limitations

The principal contribution of this case lies in the integration of pathological review, serial laboratory findings, and longitudinal imaging changes, which demonstrated a clear discrepancy between the initial benign diagnosis and the subsequent aggressive clinical course. Several limitations should be acknowledged. First, the archived pathological material could not be retrieved, precluding additional sampling and supplementary immunohistochemical studies needed to more definitively exclude a malignant littoral cell tumor. Second, the patient declined a liver biopsy; consequently, the hepatic lesions can only be regarded as radiologically highly suspicious for metastases rather than histologically confirmed metastatic disease. Finally, because the patient did not receive subsequent antitumor treatment, this case provides no evidence regarding therapeutic efficacy.

## 5. Conclusion

This case highlights the diagnostic overlap among PSAn, LCA, and malignant tumors of littoral cell origin. Although the pathological review favored PSAn, a malignant littoral cell tumor could not be entirely excluded because of the marked morphological heterogeneity and the unavailability of additional pathological material. Clinicians should promptly initiate a multidisciplinary clinicopathological review when a patient initially diagnosed with a benign splenic vascular tumor develops an aggressive postoperative course characterized by persistent fever, progressive cytopenias, worsening liver function, and new multifocal hepatic lesions.

## Acknowledgments

The authors would like to thank Editage for scientific editing of the manuscript, and the staff at the Department of Emergency Medicine at the First Affiliated Hospital of Zhejiang Chinese Medical University for their help in the management of this patient.

## Author contributions

**Conceptualization:** Yi Yu.

**Visualization:** Yi Yu.

**Funding acquisition:** Hong Zhang, Liying Xu.

**Investigation:** Hong Zhang, Gaosong Zhang.

**Resources:** Hong Zhang, Gaosong Zhang.

**Supervision:** Hong Zhang.

**Validation:** Hong Zhang.

**Methodology:** Liying Xu.

**Project administration:** Liying Xu.

**Writing – original draft:** Yi Yu, Gaosong Zhang, Liying Xu.

**Writing – review & editing:** Yi Yu, Hong Zhang, Liying Xu.




