# Supplementary material for: Primary splenic angiosarcoma initially diagnosed as littoral cell angioma: A case report of a diagnostic pitfall
Source: Medicine (Baltimore). 2026 Nov 6;105(45):e51451. doi: 10.1097/MD.0000000000051451 (PMC13747549; doi:10.1097/MD.0000000000051451)
Supplement: Supplementary file 1 [file medi-105-e51451-s002.docx]

**Supplemental Digital Content 2. Published cases relevant to the diagnostic overlap among primary splenic angiosarcoma, benign splenic vascular tumors, and littoral-cell neoplasms**

| **Reference** | **Initial diagnosis and key pathological findings** | **Final diagnosis and clinical course** | **Similarities to the present case** | **Major differences from the present case** |
| --- | --- | --- | --- | --- |
| **Group A. PSAn initially interpreted as a benign splenic vascular tumor** | | | | |
| Xu et al., 2015 | The splenectomy specimen was initially diagnosed as splenic hemangioma. The lesional cells showed only mild atypia and were positive for CD31 and CD34 but negative for factor VIII-related antigen, CD68, and p53. | Two years after splenectomy, the patient developed severe anemia and lesions involving the liver, bone marrow, and an accessory spleen. Liver and bone marrow biopsies showed findings similar to those of the original splenic lesion. Comparison of these specimens led to a revised diagnosis of well-differentiated PSAn with widespread metastases. The patient received no chemotherapy and died 3 months after discharge. The original splenic lesion was additionally analyzed using array-CGH. | The splenic lesion was initially interpreted as a benign vascular tumor. Subsequent hepatic involvement and an aggressive clinical course prompted reassessment of the original pathological diagnosis. | The initial diagnosis was hemangioma rather than LCA. Disease progression occurred after 2 years, and the hepatic and bone marrow lesions were histologically examined. Genomic analysis was also performed. |
| Cao et al., 2019 | The patient underwent splenectomy for spontaneous splenic rupture, and the postoperative diagnosis was splenic hemangioma. The original report described CD31 positivity and negative CD34 staining. | Multiple hepatic lesions were detected by CT 2 years after splenectomy. Liver biopsy was highly suggestive of metastatic PSAn. The patient underwent TACE and NGS but declined subsequent targeted therapy. She died within 51 months after splenectomy. | Hepatic progression occurred after an initial diagnosis of a benign splenic vascular tumor, leading to reconsideration of the nature of the original splenic lesion. | The initial diagnosis was hemangioma rather than LCA, and the patient initially presented with spontaneous splenic rupture. Disease progression was slower, the hepatic lesions were biopsied, and interventional treatment and genomic testing were performed. |
| **Group B. Malignant littoral-cell neoplasms containing LCA-like areas** | | | | |
| Ben-Izhak et al., 2001 | Most of the original splenic lesion showed conventional LCA-like morphology, although solid areas and small foci of necrosis were also present. The tumor cells expressed factor VIII-related antigen, CD31, CD68, and CD21 but were negative for CD34 and CD8. | Eight years after splenectomy, the patient developed an abdominal recurrence and multiple hepatic metastases. The recurrent tumor showed increased solid architecture, greater cytologic atypia, and a higher Ki-67 proliferation index. The final diagnosis was LCHE. | The case demonstrates that relatively bland LCA-like areas may obscure components with malignant potential and that subsequent hepatic disease may prompt reassessment of the original splenic lesion. | The final diagnosis was low-grade LCHE rather than PSAn. The latency period was 8 years, markedly longer than the approximately 3-month interval in the present case. |
| Fernandez et al., 2006 | The original splenic lesion predominantly showed conventional LCA-like morphology but also contained solid nests of cytologically bland clear cells. The tumor cells expressed CD31, CD68, CD21, and CD163 but were negative for CD8 and CD34. | Four years after splenectomy, hepatic and retroperitoneal metastases developed. The splenic and hepatic lesions showed identical morphological and immunophenotypic features, leading to a diagnosis of metastatic low-grade LCHE. | This case demonstrates that solid cellular areas may indicate malignant potential even in the absence of marked cytologic atypia. | The final diagnosis was LCHE rather than PSAn. The disease followed a relatively indolent course, and the metastatic lesions were histologically confirmed. |
| Larsen et al., 2013 | The splenic nodules resembled LCA but also contained solid areas composed of cytologically bland spindle cells. The tumor cells expressed CD31, factor VIII-related antigen, CD68, and CD163 but were negative for CD8 and CD34. | Morphologically and immunophenotypically similar spindle cells diffusely infiltrated the cirrhotic liver. The final diagnosis was low-grade LCAS. Pancytopenia resolved after splenectomy, and the patient remained asymptomatic at 19 months. | Both cases showed LCA-like morphology, overlapping endothelial and histiocytic marker expression, and hepatic involvement. | The final diagnosis was LCAS rather than PSAn. The patient was a 38-year-old man, and hepatic involvement manifested as diffuse infiltration and cirrhosis rather than rapidly developing multifocal nodules. The clinical course was relatively indolent. |
| Wang et al., 2013 | Preoperative splenic biopsy showed focal hemangioma-like structures without definite endothelial atypia or abnormal mitotic activity. CD34 and CD68 were positive, suggesting LCA, although angiosarcoma and LCAS could not be excluded. | The splenic and right hepatic lesions were resected simultaneously. Both consisted of anastomosing vascular channels. The tumor cells expressed CD31, CD34, broad-spectrum cytokeratin, CD68/PGM-1, and CD8 but were negative for factor VIII-related antigen and CD21; the Ki-67 index was <2%. The final diagnosis was LCAS with synchronous hepatic metastasis. No recurrence was detected during the first postoperative year. | The case showed LCA-like morphology, overlapping endothelial and histiocytic marker expression, and associated hepatic lesions. | The final diagnosis was LCAS rather than conventional PSAn. Hepatic metastasis was synchronous and histologically confirmed. The patient was a 28-year-old woman, the Ki-67 index was low, and the postoperative course was relatively favorable. |
| **Group C. Other cases providing relevant supporting evidence** | | | | |
| Takayoshi et al., 2018 | The patient had undergone splenectomy for a lesion diagnosed as LCA 10 years earlier. | Ten years after splenectomy, the patient developed bicytopenia, hepatomegaly, and multiple hepatic lesions. Liver biopsy demonstrated intrasinusoidal infiltration by macrophage-like tumor cells that expressed CD31, CD68, and CD163 but were negative for CD34, factor VIII-related antigen, ERG, hepatocellular, epithelial, and myogenic markers. The authors diagnosed metastatic LCA. The patient showed a partial response to chemotherapy, including etoposide and paclitaxel. | Both patients developed cytopenias, hepatomegaly, and multiple hepatic lesions after an initial diagnosis of LCA. | The final diagnosis remained metastatic LCA rather than PSAn or histologically defined LCHE/LCAS. The latency period was 10 years, the hepatic lesions were histologically confirmed, and systemic treatment produced a clinical response. |
| Mariz et al., 2026 | A 64-year-old woman underwent splenectomy for a heterogeneous splenic lesion complicated by capsular rupture. The tumor showed hemorrhage, necrosis, and atypical endothelial cells forming irregular vascular spaces. The tumor cells expressed ERG, CD31, CD34, CD68, and CD163, and the lesion was diagnosed directly as PSAn. The authors noted that this immunophenotype could be mistaken for LCA if cytologic atypia were less pronounced. | The report primarily addressed the morphological heterogeneity of PSAn and its pathological distinction from LCA. It did not describe an actual initial misdiagnosis as LCA followed by diagnostic revision. | The case supports the possibility that PSAn may coexpress endothelial and histiocytic markers, indicating that CD68 or CD163 positivity alone does not establish littoral-cell differentiation. | This was not a case of actual misdiagnosis or delayed diagnosis. It therefore provides supporting evidence of immunophenotypic overlap rather than a direct clinical comparator. |

**Note:** Group A directly illustrates the potential for well-differentiated or morphologically heterogeneous PSAn to be interpreted as a benign splenic vascular tumor. Group B comprises malignant littoral-cell neoplasms that are pathologically distinct from conventional PSAn but are relevant to the differential diagnosis because of their extensive LCA-like areas and overlapping endothelial–histiocytic immunophenotypes. Group C provides supplementary evidence regarding metastatic behavior or immunophenotypic overlap.

**Abbreviations:** PSAn, primary splenic angiosarcoma; array-CGH, array comparative genomic hybridization; LCA, littoral cell angioma; CT, computed tomography; TACE, transcatheter arterial chemoembolization; NGS, next-generation sequencing; LCHE, littoral cell hemangioendothelioma; LCAS, littoral cell angiosarcoma.
